# P-72 - ESTABLISHING THE BASELINE EPIDEMIOLOGY AND BURDEN OF CHICKENPOX IN SCOTLAND, PRIOR TO LAUNCH OF THE INFANT VARICELLA VACCINATION PROGRAMME

**DOI:** 10.1093/pubmed/fdag046.093

**Published:** 2026-07-09

**Authors:** Mrs Aynsley Milne, Mrs Adriana Zalewska, Dr Christopher Sullivan, Dr Daniel Chandler, Dr Cheryl Gibbons

**Affiliations:** Public Health Scotland; Public Health Scotland; Public Health Scotland; Public Health Scotland; Public Health Scotland

## Abstract

**Background:**

Chickenpox is a highly contagious childhood infection, yet its true burden in Scotland is difficult to quantify due to limited surveillance and its status as a non-notifiable disease.

**Objectives:**

Following recent recommendations for a national varicella vaccination programme, this study aims to characterise the current epidemiology and disease burden using routinely collected healthcare data to establish a baseline for future impact and effectiveness assessments.

**Methods:**

Three complementary surveillance sources were analysed: chickenpox-related hospital admissions, laboratory-confirmed cases, and GP consultations. Descriptive epidemiology was undertaken to examine secular trends in incidence, stratified by age group and deprivation quintile. Additional indicators included length-of-hospital-stay (LOS), seasonal patterns, and annual variation. Data were summarised using tables, time-series plots, and demographic breakdowns.

**Results:**

Across the study period, there were 7,309 hospital admissions (5.32 per 100,000 population over 25 years, decreasing to 4.98 per 100,000 in the most recent five years). Admission rates were highest in infants aged <1 year (86.72 per 100,000), with notable year-to-year fluctuation. A social gradient was observed, with the highest burden in the most deprived quintile (6.45 per 100,000). Median LOS was 4.61 days overall and 3.09 days for infants.

GP consultation data showed a consistent decline from 2018 to 2025, with substantial disruption during the COVID-19 pandemic. A total of 31,171 chickenpox-related consultations were recorded, with incidence highest in younger age groups and in more deprived areas.

Laboratory surveillance showed inconsistent trends: infants had lower rates, and incidence increased with age, which contradicted hospital-based findings.

**Conclusions:**

Differences across data sources highlight challenges in establishing a baseline. Reduced routine testing and variable referral practices limit the usefulness of laboratory data for surveillance, while evolving service models may influence consultation trends. Despite these limitations, GP and hospital datasets provide a robust foundation for future vaccine impact and effectiveness evaluations.

